# Trends in the Epidemiology of Deep Vein Thrombosis and Pulmonary Embolism in Patients Undergoing Surgery

**DOI:** 10.7759/cureus.74925

**Published:** 2024-12-01

**Authors:** Shelby Cowan, Kassem Ghayyad, Matthew J Conlon, Maya Naik, Ibrahim Zeini, Michael Hawks, Atif Ahmed, Amir R Kachooei

**Affiliations:** 1 Orthopaedic Surgery, Rothman Orthopaedics Florida at AdventHealth, Orlando, USA; 2 Orthopaedics, University of Central Florida, Orlando, USA; 3 Orthopaedics, Rothman Orthopaedics Florida at AdventHealth, Orlando, USA; 4 Orthopaedics, Orthopedic Research Center, Mashhad University of Medical Sciences, Mashhad, IRN

**Keywords:** deep vein thrombosis (dvt), factor v leiden deficiency, lower extremity dvt, medical comorbidities, upper extremity dvt, venous thromboembolism (vte), vte prophylaxis

## Abstract

Objective

This study aims to utilize the TriNetX database, a comprehensive global network, to improve our understanding of the frequency, demographic factors, and related comorbidities of surgical patients who develop venous thromboembolism (VTEs) events.

Methods

The global collaborative network in TriNetX was queried for all cases from January 1, 2017, through December 31, 2023. International Classification for Disease (ICD) diagnosis codes were used to define patient cohorts with deep vein thrombosis (DVT) of the upper or lower extremity or pulmonary embolism (PE). Patient information was extracted including age, sex, ethnicity, race status, and comorbidities. We assumed that PE occurred following a DVT which is only reported once as a PE, and not a DVT.

Results

The study included 414,045 patients with lower extremity DVT, 82,800 with upper extremity DVT, and 508,044 with reported PE following a DVT. DVT and PE account for approximately 51% and 49% of VTE cases, respectively, with differences noted based on age, sex, ethnicity, race, and comorbidities. The data showed that advanced age, higher BMI, and Black race are associated with a higher risk of thromboembolism. Common comorbidities, such as cardiac dysrhythmias, a history of thromboembolism, cancer, and renal failure are prevalent across all three diagnostic groups.

Conclusion

The study results suggest that the incidence and prevalence of VTE are changing due to the aging population and changes in demographic patterns. Healthcare services should consider planning for the changes in morbidity, mortality, and related healthcare costs. Surgical patients with multiple related comorbidities should be managed to prevent VTEs more aggressively with close monitoring for any evolving VTE.

## Introduction

A venous thromboembolism (VTE) event involves a blood clot lodged in the extremities' deep veins or branches of the pulmonary artery. Deep vein thrombosis (DVT) can occur in either the upper or lower extremities, and pulmonary embolism (PE) is most commonly a result of a DVT that travels through the circulation from the lower extremities [1]. Virchow’s Triad identifies three overarching risk factors that contribute to the development of thromboembolism: venous stasis, endothelial injury, and a hypercoagulable state. All surgeries increase the risk of thromboembolism, but major orthopedic surgeries can cause patients to be at particularly elevated risk, due to the significant impact on all three factors. It is estimated that 4% of patients who undergo major orthopedic surgeries to the lower extremities develop DVT or PE, with the risk being greatest in the first 7-14 days post-op [2].

Thromboembolic complications perioperatively can lengthen a patient’s hospital stay, result in additional healthcare costs, and increase mortality [3]. VTE, including cases that occur postoperatively and from other causes, accounts for 60,000-100,000 deaths each year in the United States, most commonly resulting from a PE that forms on its own or secondary to a DVT [1]. When considering surgery, it is important to consider demographic factors and comorbidities that may further elevate a patient’s risk of developing a thrombus to implement a more aggressive approach to monitoring and preventing VTEs. The use of preventative interventions like mechanical devices to prevent venous stasis and, primarily, pharmacologic anticoagulants must be balanced with the risk of major bleeding events that can occur as a result of surgery, making it essential to identify risk factors that would warrant more or less aggressive prophylaxis [2-4].

This study aims to leverage the TriNetX database, an extensive global network, to enhance our comprehension of the frequency, demographic variables, and associated comorbidities of patients undergoing surgery who develop DVT or PE.

## Materials and methods

A retrospective study using TriNetX global Collaborative Network was performed and queried on May 14, 2024. The methodology of data collection does not include patient-identifiable information and is thus exempt from the Institutional Review Board (IRB). The patient cohorts were defined using the International Classification for Disease, 10th Edition (ICD-10) diagnosis codes. All patients diagnosed with DVT of the upper or lower extremity or PE between January 1, 2017, and December 31, 2023, were included.

Patient cohorts were subsequently defined by inclusion and exclusion of DVT or PE through utilization of the diagnostic (ICD-10) system codes. The lower extremity DVT group (LE DVT) contained ICD-10 codes for acute embolism and thrombosis of unspecified deep veins of the right or left lower extremity (182.401, 182.402) or unspecified lower extremity (182.40, 182.409). The upper extremity DVT group (UE DVT) contained ICD-10 codes for acute embolism and thrombosis of unspecified deep veins of the right, left, or unspecified upper extremity (182.621, 182.622, 182.62, 182.629) The PE group contained ICD-10 codes for PE without cor pulmonale (126.9, 126.99).

Data extracted

Patient information was extracted including age, sex, ethnicity, race status, and comorbidities. Age was divided into four quartiles including 0-17, 18-39, 40-64, and 65-90 years of age. Ethnicity was divided into Hispanic or Latino, Not Hispanic or Latino, and unknown. Race was divided into White, Black, Asian, Native American or Pacific Islander, American Indian or Alaska Native, other, and unknown. Comorbidities were recorded based on ICD-10 codes.

Global Collaborative Network

The Global Collaborative Network through TriNetX is a web-based database tool that allows for research of population cohorts, feasibility queries, and collaboration with medical researchers worldwide. The database contains a large network of over four hundred million de-identified patient data that can be accessed on demand without prior IRB approval. The database allows access to patient demographics, diagnoses, procedures, labs, and medications. Data is obtained through collaboration between over two hundred community and academic-based healthcare organizations and industry partners worldwide.

## Results

Patient demographics

**Figure 1:**
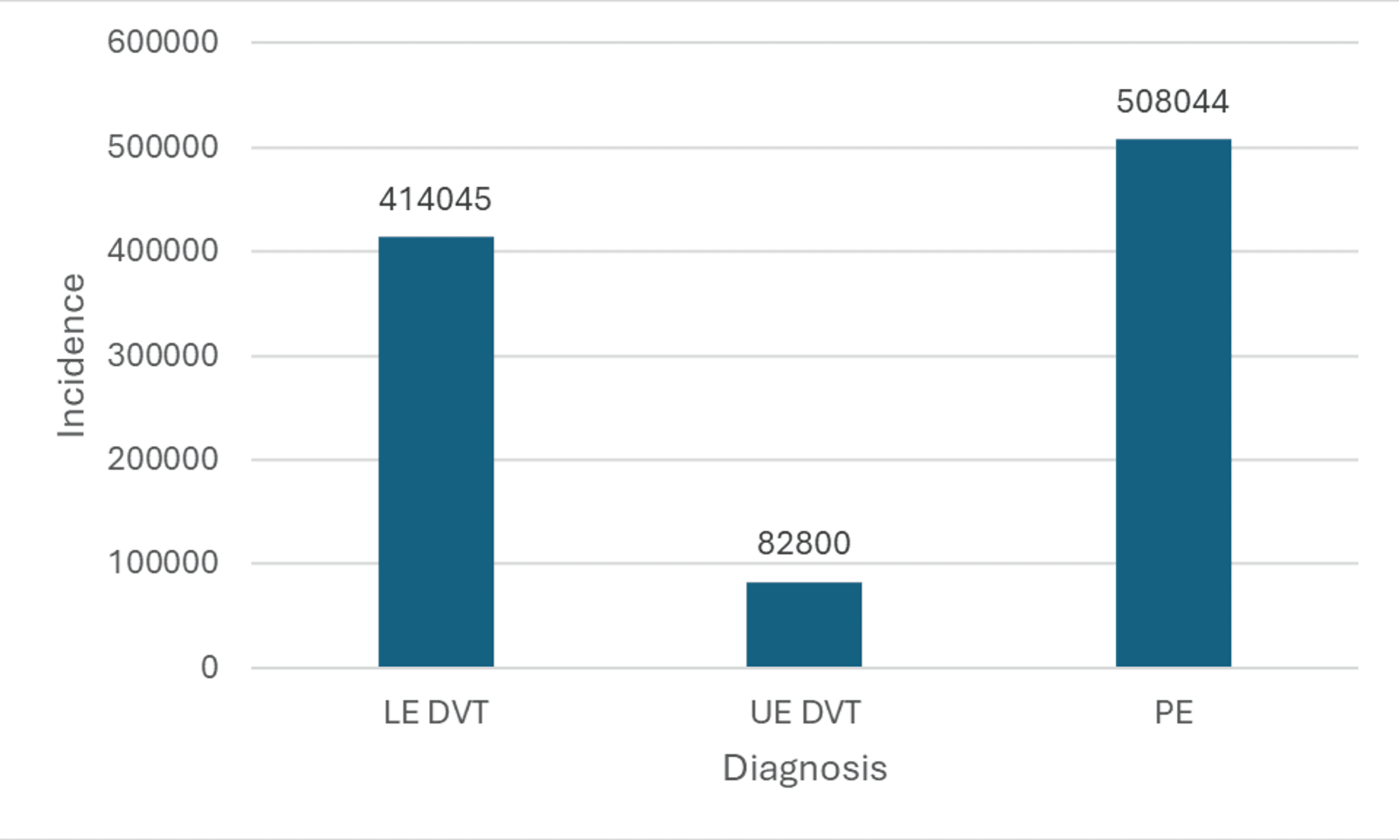
Bar Graph Showing Incidence of Lower Extremity Deep Vein Thrombosis (LE DVT), Upper Extremity Deep Vein Thrombosis (UE DVT), and Pulmonary Embolism (PE) From 2017 to 2023

Since the PEs mainly occurred after a DVT, the DVT and subsequent PE are counted as a single VTE towards a PE. A total of 414,045 patients with LE DVT, 82,800 patients with UE DVT, and 508,044 patients with PE were recorded between 2017 and 2023 (Table 1 and Figure 1).

**Table 1:** Total Cases of Deep Vein Thrombosis and Pulmonary Embolism (2017-2023) With Demographic Breakdown LE DVT: lower extremity deep vein thrombosis group; UE DVT: upper extremity deep vein thrombosis group; PE: pulmonary embolism

Characteristics	LE DVT	UE DVT	PE
Total Patients	4,14,045	82,800	5,08,044
Age			
0-17	2103 (0.5%)	1191 (1.4%)	1131 (0.2%)
18-39	31551 (7.6%)	11491 (13.9%)	48535 (9.6%)
40-64	138944 (33.6%)	30645 (37.0%)	172381 (33.9%)
65-90	241447 (58.3%)	39473 (47.7%)	285997 (56.3%)
Sex			
Female	198662 (48.0%)	38536 (46.5%)	252209 (49.6%)
Male	192284 (46.4%)	39747 (48.0%)	229278 (45.1%)
Unknown	23099 (5.6%)	4517 (5.5%)	26557 (5.2%)
Ethnicity			
Hispanic or Latino	23525 (5.7%)	6379 (7.7%)	25318 (5%)
Not Hispanic or Latino	291480 (70.4%)	58626 (70.8%)	357735 (70.4%)
Unknown	99040 (23.9%)	17795 (21.5%)	124991 (24.6%)
Race			
White	277637 (67.1%)	50212 (60.6%)	327098 (64.4%)
Black	56181 (13.6%)	16288 (19.7%)	86234 (17%)
Other	11044 (2.7%)	2749 (3.3%)	14766 (2.9%)
Asian	6919 (1.7%)	1789 (2.2%)	8272 (1.6%)
Native Hawaiian or Other Pacific Islander	1108 (0.3%)	275 (0.33%)	1302 (0.3%)
American Indian or Alaskan Native	942 (0.2%)	217 (0.26%)	1260 (0.2%)
Unknown	60214 (14.5%)	11270 (13.6%)	69012 (13.6%)

From 2017 to 2023, 508,044 (51%) of the VTEs are reported as PE. The ratio of a non-PE DVT of the lower to upper extremities is 5:1. However, considering most of the PEs occurred after LE DVT, the ratio of lower to upper extremity DVT is assumed to be nearly 11:1.

**Figure 2:**
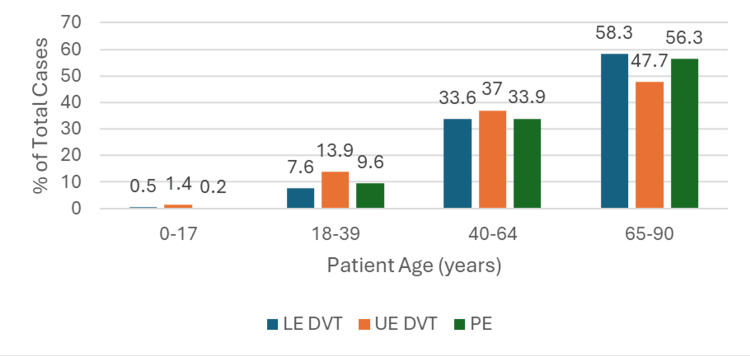
Bar Graph Showing Proportion of Lower Extremity Deep Vein Thrombosis (LE DVT), Upper Extremity Deep Vein Thrombosis (UE DVT), and Pulmonary Embolism (PE) Cases by Age Group

With regard to age, all three diagnostic groups followed the same trend; the incidence of VTE increased with age. The highest proportion of cases were seen in the 65-90 group, representing 241,447 (58%) of LE DVT, 39,473 (48%) of UE DVT, and 285,997 (56%) of PE cases (Table 1). The 0-17 age group made up the smallest proportion of cases, ranging from 1,131 (0.2%) of PEs to 1,191 (1.4%) of UE DVTs (Figure 2). 

**Figure 3:**
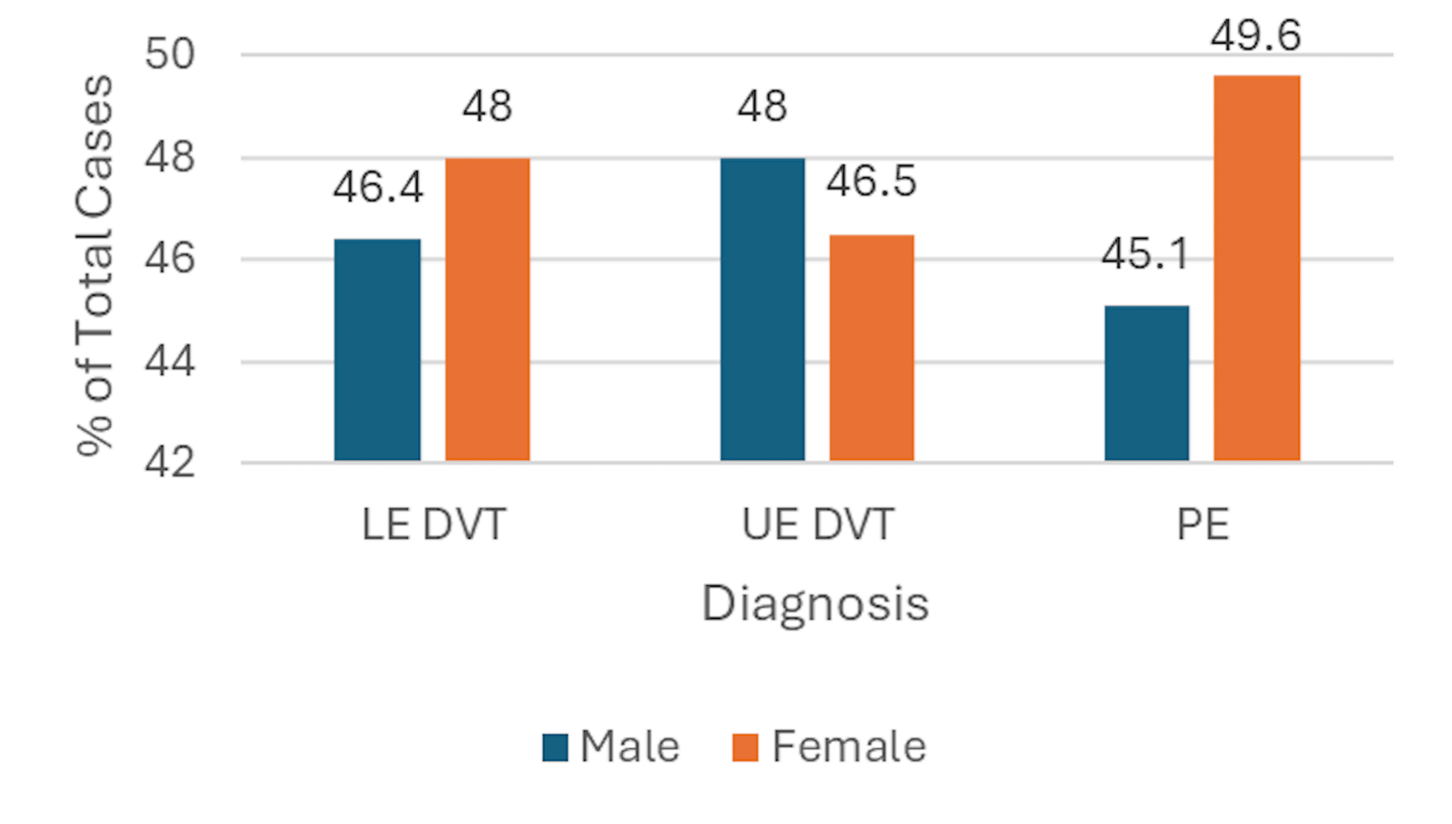
Bar Graph Showing the Percentage of Lower Extremity Deep Vein Thrombosis (LE DVT), Upper Extremity Deep Vein Thrombosis (UE DVT), and Pulmonary Embolism (PE) Cases by Sex

When considering sex, slightly more cases of LE DVT and PE were reported in females than in males (Table 1). The female:male ratio in the LE DVT group was 1.03:1 and was 1.10:1 in the PE group. In contrast, more cases of UE DVT were seen in males; a female:male ratio of 0.97:1 was observed (Figure 3).

**Figure 4:**
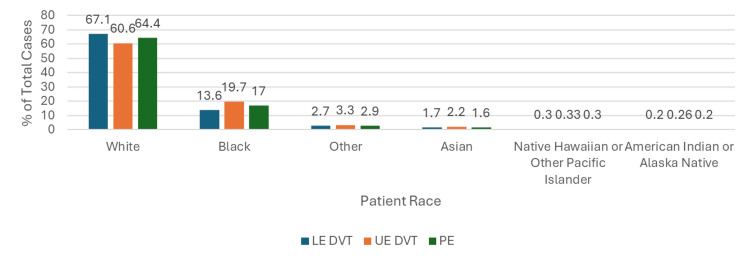
Bar Graph Showing the Percentage of Lower Extremity Deep Vein Thrombosis (LE DVT), Upper Extremity Deep Vein Thrombosis (UE DVT), and Pulmonary Embolism (PE) Cases by Race

When examining patient race, VTEs were reported most commonly in the White race comprising between 50,212 (61%) in the UE DVT group and 277,637 (68%) in the LE DVT group, followed by the Black race comprising between 56,181 (14%) in LE DVT and 16,288 (20%) of events in UE DVT group (Figure 4).

Patient comorbidities

Regarding BMI, there is a considerable proportion of patients in all three diagnostic groups who are considered obese with a BMI >30.0, representing between 25,507 (31%) and 136,164 (33%) of patients in UE DVT and LE DVT, respectively. A BMI of 19.9 or below, indicating an underweight status, was uncommon in any diagnostic group.

**Figure 5:**
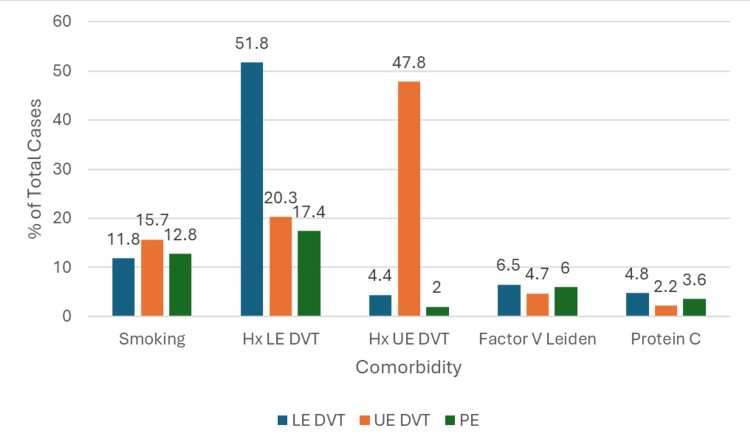
Bar Graph Depicting the Proportion of Patients With Lower Extremity Deep Vein Thrombosis (LE DVT), Upper Extremity Deep Vein Thrombosis (UE DVT), and Pulmonary Embolism (PE) Who Have Comorbidities Related to Coagulation

When considering comorbidities that relate to coagulation, a history of previous DVT is highly predictive. It was reported that 39,546 (48%) of UE DVT patients had a previous thrombus in an upper extremity, and 214,673 (52%) of LE DVT patients had a previous thrombus in a lower extremity (Figure 5).

**Figure 6:**
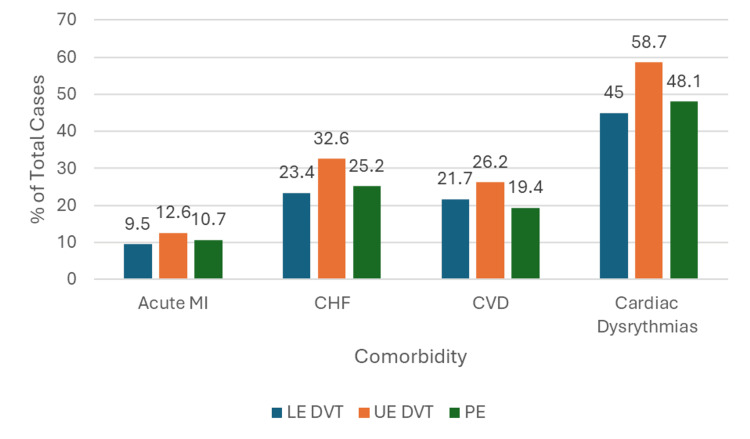
Bar Graph Showing the Percentage of Lower Extremity Deep Vein Thrombosis (LE DVT), Upper Extremity Deep Vein Thrombosis (UE DVT), and Pulmonary Embolism (PE) Patients With Certain Cardiovascular Comorbidities

Cardiovascular comorbidities were prevalent among patients with thromboembolic diagnoses. Cardiac dysrhythmias were reported in 186,329 (45%) of LE DVT, 244,172 (48%) of PE, and 48,585 (59%) of the UE DVT events. Congestive heart failure (CHF) was reported in 27,009 (33%) of the UE DVT events. Cardiovascular disease (CVD) is reported in between 98,697 (19%) and 21,700 (26%) patients, again being most common among UE DVT events (Figure 6).

**Figure 7:**
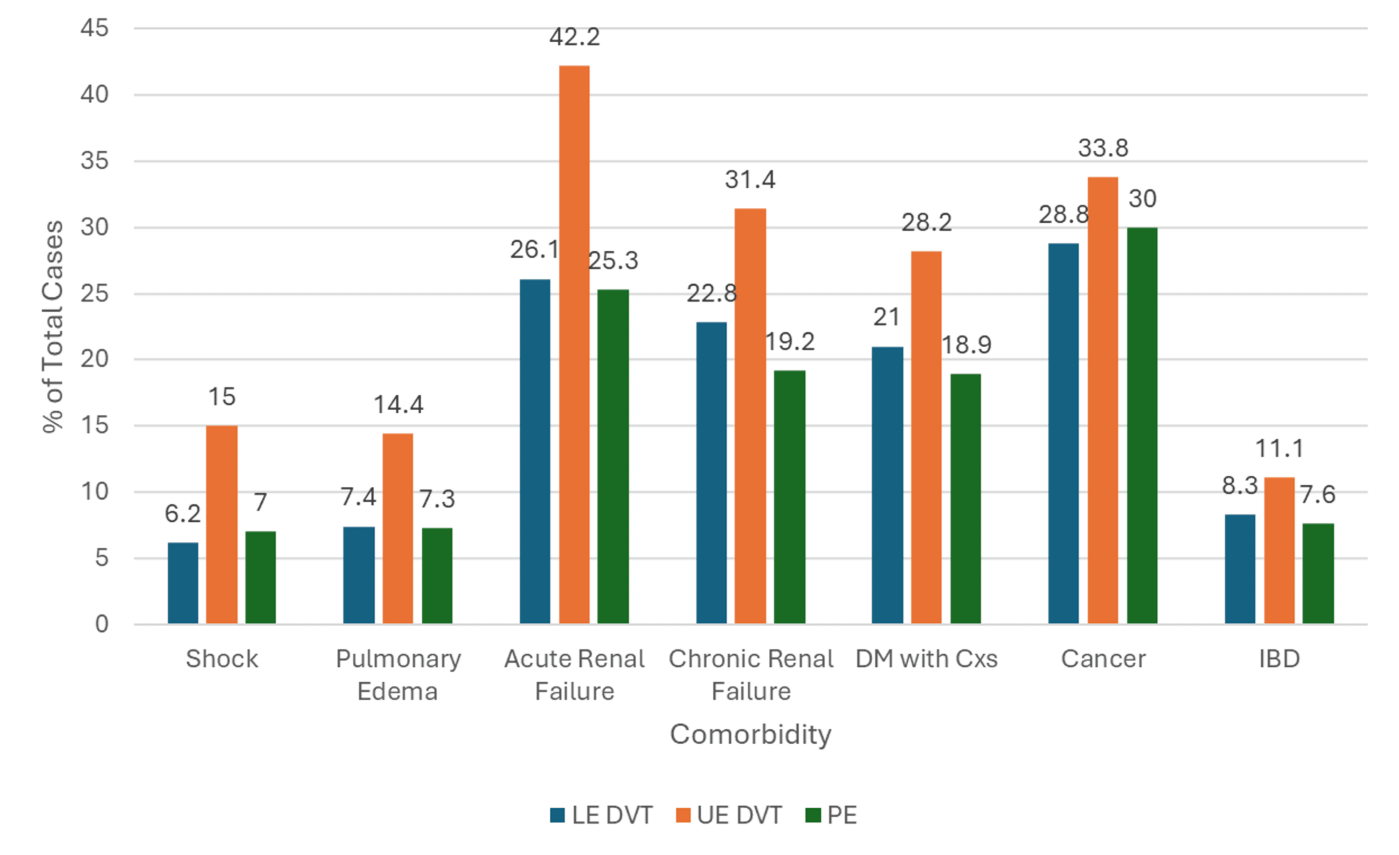
Bar Graph Showing the Proportion of Lower Extremity Deep Vein Thrombosis (LE DVT), Upper Extremity Deep Vein Thrombosis (UE DVT), and Pulmonary Embolism (PE) Patients With Additional Comorbidities DM with Cxs: diabetes mellitus with complications; IBD: inflammatory bowel disease

When considering other comorbidities, acute renal failure is most commonly reported with UE DVT comprising 34,912 (42%) of the events, while it is reported in 107,986 (26%) of LE DVT and 128,659 (25%) of PE events. Cancer comorbidity was reported among LE DVT, UE DVT, and PE patients, representing 119,049 (29%), 27,978 (34%), and 152,269 (30%), respectively (Figure 7).

Complete comorbidity data is shown in Table 2.

**Table 2:** Percentage of Patients in Each Diagnostic Group With Selected Comorbidities LE DVT: lower extremity deep vein thrombosis group; UE DVT: upper extremity deep vein thrombosis group; PE: pulmonary embolism; MI: myocardial infarction; CHF: congestive heart failure; CVD: cerebrovascular disease; DM with Cxs: diabetes mellitus with complications; IBD: inflammatory bowel disease

Comorbidity	LE DVT	UE DVT	PE
BMI			
Obesity	136164 (32.9%)	25507 (30.8%)	165988 (32.7%)
BMI 19.9	11521 (2.8%)	4760 (5.7%)	16498 (3.2%)
Clotting			
Smoking	48924 (11.8%)	13031 (15.7%)	65186 (12.8%)
History DVT LE	214673 (51.8%)	16817 (20.3%)	88500 (17.4%)
History DVT UE	18181 (4.4%)	39546 (47.8%)	10250 (2.0%)
Factor V Leiden (D68.59)	27056 (6.5%)	3898 (4.7%)	29938 (6.0%)
Protein C	20045 (4.8%)	1819 (2.2%)	18449 (3.6%)
Pregnancy	3836 (0.9%)	926 (1.1%)	5385 (1.1%)
Initial Contraceptive Rx	627 (0.2%)	174 (0.2%)	1017 (0.2%)
Surveillance Contraceptive	770 (0.2%)	203 (0.2%)	1120 (0.2%)
Emergency Contraceptive	53 (0.01%)	17 (0.02%)	96 (0.02%)
Cardiovascular			
Acute MI	39469 (9.5%)	11247 (13.6%)	54129 (10.7%)
CHF	96736 (23.4%)	27009 (32.6%)	127815 (25.2%)
CVD	90049 (21.7%)	21700 (26.2%)	98697 (19.4%)
Cardiac Dysrhythmias	186329 (45.0%)	48585 (58.7%)	244172 (48.1%)
Other			
Shock	25614 (6.2%)	12388 (15.0%)	35315 (7.0%)
Pulmonary Edema	30622 (7.4%)	11950 (14.4%)	37088 (7.3%)
Acute Renal Failure	107986 (26.1%)	34912 (42.2%)	128659 (25.3%)
Chronic Renal Failure	94589 (22.8%)	25991 (31.4%)	97345 (19.2%)
DM With Cxs	87071 (21.0%)	23317 (28.2%)	96077 (18.9%)
Cancer	119049 (28.8%)	27978 (33.8%)	152269 (30.0%)
IBD	34455 (8.3%)	9180 (11.1%)	38567 (7.6%)
Surgical Procedure	1513 (0.4%)	513 (0.6%)	1623 (0.3%)
Mobility			
Paraplegic	444 (0.1%)	110 (0.1%)	407 (0.1%)
Short Leg Cast	2538 (0.6%)	452 (0.5%)	2296 (0.5%)
Long Leg Cast	1349 (0.3%)	264 (0.3%)	1494 (0.3%)

## Discussion

Surgery, in and of itself, increases a patient’s risk of developing DVT or PE, so it is crucial to identify groups that are particularly susceptible and tailor preventative interventions accordingly. This study presents insight into demographic variables and comorbid conditions that were present in patients who developed a thromboembolic event between 2017 and 2023. Differences in the prevalence of DVT and PE were found overall and when considering age, sex, and race. In addition, comorbid conditions that affect blood coagulation, the cardiovascular system, and other systemic conditions were reported with varying prevalence in this population.

The use of the TriNetX database to obtain patient data allowed for a vast and heterogeneous sample of patients, reducing the likelihood of confounding factors in this study. However, there are some limitations; there was incomplete demographic information on some patients, the type of surgery was not reported, and the data was not presented in a way that allowed for examination of how demographic factors or multiple comorbidities intersected to increase risk. Additionally, the geographic distribution of this data was not available, so it is unclear how differential reporting in certain areas may have affected the results. Consequently, the results of this study should not be used to conclude but instead should be used as guidance for further analysis. We have published two previous epidemiological studies using the TriNetX database [5,6].

About half of all VTEs were reported as PE. Considering that a PE after a DVT is only reported as a PE, the number of DVTs would be doubled, showing that nearly half of the DVTs may result in a PE. However, previous studies of the general population have shown that PE occurs as a complication of UE and LE DVT in 6% and 15%-30% of cases, respectively, lower than expected from Grigorian and Nahmias' study [7]. Additionally in the general population, UE DVT appears to make up 5%-10% of all DVT cases [7], lower than the 17% of cases in this study. UE DVT has been reported in association with orthopedic surgery, specifically arthroscopic procedures of the shoulder, so postoperative cases may also explain this higher prevalence [8].

There was no significant difference in the number of thromboembolic cases reported in males versus females, but more cases of LE DVT and PE were reported in females, while more UE DVT were reported in males. In the general population, Scheres et al. reported in 2022 that primary cases of DVT and PE occur equally in males and females throughout the lifespan. However, incidence rates are higher in females before menopause, around age 50, and after age 70. Men have a higher incidence rate than females between 50 and 70 years of age, and the reoccurrence of thromboembolism occurs at a rate twice as high in males compared to females. Males appear to have a higher intrinsic risk of thromboembolism overall, because when female-specific reproductive factors such as contraceptives and pregnancy are corrected, men exhibit a higher incidence rate [9]. In this study, a very small proportion of patients used contraceptives or were pregnant at the time of thromboembolism, so the higher incidence of UE DVT in males follows the aforementioned trends. While the oldest age group in this study begins at age 65 rather than 70, the age noted in the literature when thromboembolism incidence is once again higher in females, the high proportion of LE DVT and PE cases that occur in the 65-90 age group may explain why the incidence of these diagnoses is slightly higher in females.

Concerning race, between 50,212 (60.6%)-277,637 (67.1%) and 56,181 (13.6)-16,288 (19.7%) of cases of all three diagnoses occurred in patients who identified as White and Black people, respectively. However, according to the 2020 US Census, 75.5% of the country’s population identifies as White people and 13.6% of the population identifies as Black people [10]. When comparing the proportion of thromboembolic cases made up by people of these races to their proportions of the general population, this study suggests that White people may be at lower risk for thromboembolism, while Black people may be at higher risk. In 2014, Zakai et al. found that DVT cases followed this trend, but there was no racial association seen with PE [11]. In this study, however, the proportion of total cases accounted for by Black patients was highest in the UE DVT and lowest in the LE DVT groups. This study does not account for region, comorbidities, or socioeconomic status as in Zakai et al., so a conclusion cannot be drawn on race alone. In addition, the US population is made up of around 6.3% Asians [10], but their contribution to the total number of thromboembolic cases was much lower, supporting a 2004 study that found the incidence of thromboembolism to be lower in a Chinese population when compared to Caucasians [12].

Regarding comorbidities that impact coagulation, the most prominent risk factor for UE DVT and LE DVT was a previous history of the respective condition. Previous studies have classified thromboembolism as a chronic condition, stating that a previous thrombus elicits a 2.1-fold increase in a patient’s future risk [4]. Smoking history was documented in 48,924 (11.8%)-13,031 (15.7%) of patients, which has been shown to increase a person’s risk of thromboembolism by 17% [13]. Less than 10% of patients in this study had genetic pro-thrombotic mutations like Factor V Leiden or protein C deficiency, but the prevalence of these conditions was higher in this study than that of the general population, supporting their well-documented correlation with thromboembolism [1]. The prevalence of Factor V Leiden varies by race, but its highest prevalence is in White people at 5% [14]; between 3,898 (4.7%) and 27,056 (6.5%) of patients in this study had this diagnosis, including all races. Protein C deficiency has a prevalence of between 0.2% and 0.5% in the US population [15], but between 1,819 (2.2%) and 20,045 (4.8%) patients in the study were reported with this.

When considering cardiovascular comorbidities, cardiac dysrhythmias were highly prevalent in all three groups; dysrhythmia was reported in between 186,329 (45%) and 48,585 (58.7%) of cases and was most prevalent among patients with UE DVT. The association between atrial fibrillation, specifically, and VTE has been well described, with PE being the most common thromboembolic event in these patients [16]. CHF was also prevalent in all three diagnostic groups; it was most prevalent among UE DVT and least prevalent among LE DVT patients. A 2021 meta-analysis found that individuals with CHF have a significantly increased long-term risk for PE but not DVT; although, it was noted that DVT outside of the lower extremities was more likely to be missed by diagnostic tools used in these studies, and a higher prevalence of CHF has been seen in patients with UE DVT compared to LE DVT [17].

Comorbidities such as cancer, obesity, and chronic renal failure have been well-documented as factors that increase a patient’s risk for thromboembolism, and the results of this study showed a high prevalence of all three [4]. Although much less studied, acute renal failure has been documented as an independent risk factor for DVT and PE, citing potentially similar mechanisms to chronic kidney failure, like increased pro-coagulant proteins and endothelial injury [18]. Between 128,659 (25.3%) and 34,912 (42.2%) of patients in this study had acute renal failure. Between 96,077 (18.9%) and 23,317 (28.2%) of patients in this study had diabetes mellitus, but this is likely not a significant contributor to the development of thromboembolism, because the general population has a similarly high prevalence, and previous studies have found little association with the development of DVT or PE [19,20]. Overall, the UE DVT group had the highest incidence of all cardiovascular and miscellaneous comorbidities when compared to the other diagnostic groups. This is likely explained by the rare nature of UE DVT; multiple, cumulative risk factors are likely needed to impact Virchow’s triad to a degree great enough to elicit a thrombus in the upper extremities. Also, patients with other comorbidities may be more likely to need central catheterization perioperatively, which is a very well-documented risk for the development of UE DVT [7,8].

Orthopedic surgery is widely performed on patients of all ages. Older people, who already have a higher risk for thromboembolism and are likely to have one or more comorbid conditions, may require scheduled surgeries, like joint arthroplasty due to osteoarthritis, or more emergent procedures like fracture repairs after a fall or other trauma. Also, orthopedic surgery often requires joint immobilization and reduction in weight-bearing activities, which creates a high level of venous stasis, one of the key risks included in Virchow’s triad [2].

Prophylactic measures are critically important for patients undergoing surgical procedures. There are both mechanical and pharmacological interventions that can be used in conjunction or independently to prevent VTE. Mechanical methods include graduated compression stockings, intermittent pneumatic compression devices, and venous foot pumps. Pharmacologic interventions include aspirin, injectable low molecular weight heparin, and oral anticoagulants like Factor Xa inhibitors. There is debate as to which drug should be used for patients undergoing specific procedures or with certain comorbidities, so prophylaxis is very individualized [4]. On the other hand, orthopedic surgery creates a high risk of major bleeding events, occurring in 2-4% of cases, which must be balanced with the use of anti-thrombotic measures [4]. The International Consensus Meeting was called on this topic and recommended in 2023 that all patients undergoing major lower extremity surgery, without a normal risk for bleeding, should receive pharmacologic prophylaxis, optionally with mechanical measures [4]. For patients with a high risk of bleeding, mechanical means should be used independently. Additionally, early mobilization and initiation of physical therapy can be used to help prevent thromboembolism. Lei et al. found a significant reduction in the development of DVT in patients who began ambulating within 24 hours post-op from total knee arthroplasty [21].

## Conclusions

Over the past seven years, the rate of thromboembolism and the methods of surgical management have remained stable. Nevertheless, with an aging population, a rise in cases of DVT or PE is anticipated. This study highlights the critical need for consideration of risk factors and initiation of patient-specific preventative measures, both mechanical and pharmacological, for individuals undergoing surgeries to reduce the risk of thromboembolism.
